# Changing gloves during cesarean section for prevention of postoperative infections: a systematic review and meta-analysis

**DOI:** 10.1038/s41598-021-84259-w

**Published:** 2021-02-25

**Authors:** Siwanon Rattanakanokchai, Nuntasiri Eamudomkarn, Nampet Jampathong, Bao-Yen Luong-Thanh, Chumnan Kietpeerakool

**Affiliations:** 1 Department of Epidemiology and Biostatistics, Faculty of Public Health, Khon Kaen University, Khon Kaen, 40002 Thailand; 2 Department of Obstetrics and Gynecology, Faculty of Medicine, Khon Kaen University, Khon Kaen, 40002 Thailand; 3 Department of Epidemiology, Biostatistics and Demography, Faculty of Public Health, Hue University of Medicine and Pharmacy, Hue University, 06 Ngo Quyen Street, Hue, 530000 Vietnam

**Keywords:** Health care, Medical research

## Abstract

This systematic review and meta-analysis was conducted to assess associations between changing gloves during cesarean section (CS) and postoperative infection. A literature search was conducted using the major electronic databases MEDLINE, Scopus, ISI Web of Science, PubMed, CINAHL, and CENTRAL from their inception to September 2020. Randomized controlled trials (RCTs) comparing glove change during CS to no glove change were included. Outcomes of interest were endometritis, febrile morbidity, and incisional surgical site infection (SSI). GRADE approach was applied to assess the quality of evidence. Ten reports of six studies involving 1707 participants were included in the analyses. Glove change was associated with a reduction in the risk of incisional SSI following CS (pooled RR 0.49, 95% CI 0.30, 0.78; moderate quality of evidence). Compared to no glove change, glove change during CS did not reduce the risks of endometritis (pooled RR 1.00, 95% CI 0.80, 1.24; low quality of evidence) or febrile morbidity (pooled RR 0.85, 95% CI 0.43, 1.71; very low quality of evidence). Changing gloves during CS was associated with a decreased risk of incisional SSI. The risks of postoperative endometritis and febrile morbidity were not altered by changing gloves.

## Introduction

Cesarean section (CS) is one of the most common surgical procedures worldwide^1^. CS can be a life-saving procedure for pregnant women, fetuses, or both in certain events, including obstructed labor, abnormal placentation, obstetric hemorrhage, distressed fetus, and abnormal fetal presentation or position^1^.

For any major surgical procedure, CS may be accompanied by several postoperative complications, such as endometritis, postoperative febrile morbidity, and surgical site infection (SSI)^2^. Infectious complications are among the common short-term complications following CS^2^. The rate of infectious complications following CS varies widely from 3% to 15% depending on the risk factors for the population assessed and perioperative management^2^. Certain factors increase the risk of infectious complications following CS, including young or advanced maternal age, obesity, diabetes mellitus, previous history of CS, pre-existing genital tract infection, preterm rupture of membranes, a greater number of vaginal examinations, a prolonged trial of labor before CS, and chorioamnionitis^2^. Some of these risk factors, such as maternal obesity, pregestational diabetes mellitus, or a history of previous CS, are increasing rapidly among pregnant women worldwide, which in turn tends to increase the overall incidence of infectious complications following CS^3–5^.

The increased cost of care has been linked to post-CS infection^6^. In a previous study conducted in the US, the attributable total hospital cost of SSI and endometritis diagnosed after CS varied from $2852 to $3529 and $3842 to $3956, respectively, depending on the method applied for assessment^6^. Effective interventions for preventing infectious complications after CS, therefore, are needed.

Microbiological contamination of gloves during surgery is not uncommon^7,8^. Contamination of the surgeon’s glove with pathogenic organisms during CS may contribute to postoperative infectious morbidity^8^. Glove change during CS, therefore, may mitigate the risk of infectious complications following CS. This study was undertaken to assess whether changing gloves during CS is effective for minimizing the risk of postoperative infectious morbidity through a systematic review of randomized controlled trials.

## Methods

This systematic review was performed and is reported according to the Preferred Reporting Items for Systematic Reviews and Meta-Analyses (PRISMA) statement (Supplementary Table S1)^9^. The protocol for this systematic review was initially designed by the researcher team at Sheffield Teaching Hospitals NHS Trust, UK, and registered at PROSPERO repository on 26 October 2018^10^. The registration page did not include any updates at the time of the onset of our study; we considered the topic important because of the ease and availability of this intervention in clinical setting in LMICs and therefore decided to perform the review using revised meta-analysis methods.

### Criteria for considering studies for this review

Randomized controlled trials (RCTs) investigating a change in gloves versus no change in gloves were included regardless of the language of publication, publication status, year of publication, or sample size. The population was pregnant women who underwent CS. The CS can be a planned (elective) procedure or performed in an emergency. The intervention of interest was glove change during CS. Glove change may be performed by removing the glove and then donning a new pair of sterile gloves or by replacing the outer surgical gloves with a new pair of gloves.

This review included unpublished trials (i.e., conference proceedings, conference abstract) only if trial data and methodological descriptions were provided in written form or obtained the full report through direct contact with study authors.

### Search methods for the identification of studies

To identify potential eligible studies, a systematic literature search was conducted using the major electronic databases MEDLINE, Scopus, ISI Web of Science, PubMed, CINAHL, and Cochrane Central Register of Controlled Trials (CENTRAL) from their inception to September 10, 2020 (Supplementary Table S2). Reference lists of articles were retrieved through the search, and authors of the trials were contacted to obtain additional data if necessary. In addition, ClinicalTrials.gov and the World Health Organization International Clinical Trials Registry Platform (http://www.who.int/ictrp) were searched for unpublished, planned, and ongoing trial reports. Open Grey (http://www.opengrey.eu) was searched for grey literature. The titles of all relevant articles were identified on Google Scholar, and then a further search was performed related to these studies focusing on the first 50 records identified^11^.

### Study selection and data extraction

Titles and abstracts of studies retrieved by electronic searching were screened independently by two review authors. Studies whose titles and abstracts did not meet the inclusion criteria were excluded. The full texts of potentially eligible studies were retrieved and independently assessed by two review authors. The risk of bias of the included studies was independently evaluated by two authors using the Cochrane Risk of Bias Tool for Randomized Controlled Trials^12^. Data were extracted onto a data abstraction form specifically designed for the review. Any disagreements were resolved through discussion with a third person. We collated multiple reports of the same study so that each study rather than each report was the unit of interest. This study included the relevant intervention groups in pairwise comparisons of intervention groups that met the criteria for including studies in the review. We combined groups to create a single pairwise comparison.

The outcomes of interest were endometritis, febrile morbidity, and incisional SSI. Endometritis was defined as a body temperature ≥ 38.0 °C  (100.4 °F), fundal tenderness, and purulent discharge from the uterus. The criterion for the diagnosis of postoperative febrile morbidity was defined by the study authors, but most definitions were a temperature ≥ 38 °C following the procedure, excluding the first 24 h postoperatively. SSI was defined according to the Centers for Disease Control and Prevention’s National Healthcare Safety Network Criteria for Surgical Site Infection^13^**.** Incisional SSI assessed in this review included either superficial or deep incisional SSI.

### Data analysis

The random-effects model with Mantel–Haenszel weighting was applied for all two-level meta-analyses to calculate the risk ratios (RRs) and their 95% confidence intervals (CIs)^14^. For studies where no events were observed in one arm, a fixed value of 0.5 was added to all cells of the results table. In the study that did not use one single outcome variable but instead reported the effect of the intervention on different variables, a three-level meta-analysis was applied to avoid an overestimate of confidence in the pooled results^15,16^. We analyzed three-level meta-analyses by using a restricted maximum likelihood estimation method (REML) for estimating the parameters in the model and executed without any covariates. We presented effect estimates of three-level meta-analyses with pooled RRs and their 95% CIs.

Statistical heterogeneity in the two-level meta-analysis was assessed using the I^2^ statistic and chi-square test with a significance level of 0.10. For the three-level meta-analysis, three sources of variance were modeled: the sampling variance of the primary studies (level 1), the variance between effect sizes within studies (level 2), and the variance between studies (level 3). We applied a one-sided log-likelihood ratio test to examine heterogeneity within studies and between studies with a significance level of 0.10. We also presented the quantification of variance within-study heterogeneity (${\sigma }_{2}^{2}$) and between-study heterogeneity (${\sigma }_{3}^{2}$).

The two-level meta-analysis was performed using RevMan software version 5.4^17^. The three-level meta-analysis was conducted using the metafor package^18^ of the R statistical software^19^. Steps of a three-level meta-analysis were followed as previously recommended^20^.

### Sensitivity and subgroup analysis

Subgroup analysis was carried out according to the types of placental delivery and time of glove change. Sensitivity analysis was conducted by repeating the analysis excluding studies judged to be at a high or unknown risk of selection bias.

### Quality of evidence

We used the GRADE approach to assess the quality of evidence for each outcome. The GRADE approach covers five domains: (1) risk of bias in the included studies, (2) inconsistency between studies, (3) imprecision in the effect estimate, (4) indirectness of evidence and (5) publication bias. The GRADE approach rates the overall certainty of evidence as high, moderate, low, or very low quality^21^.

### Ethical approval

An institutional review board for ethical approval was not required for this study of deidentified information available in the public domain through prior publications.

## Results

**Figure 1:**
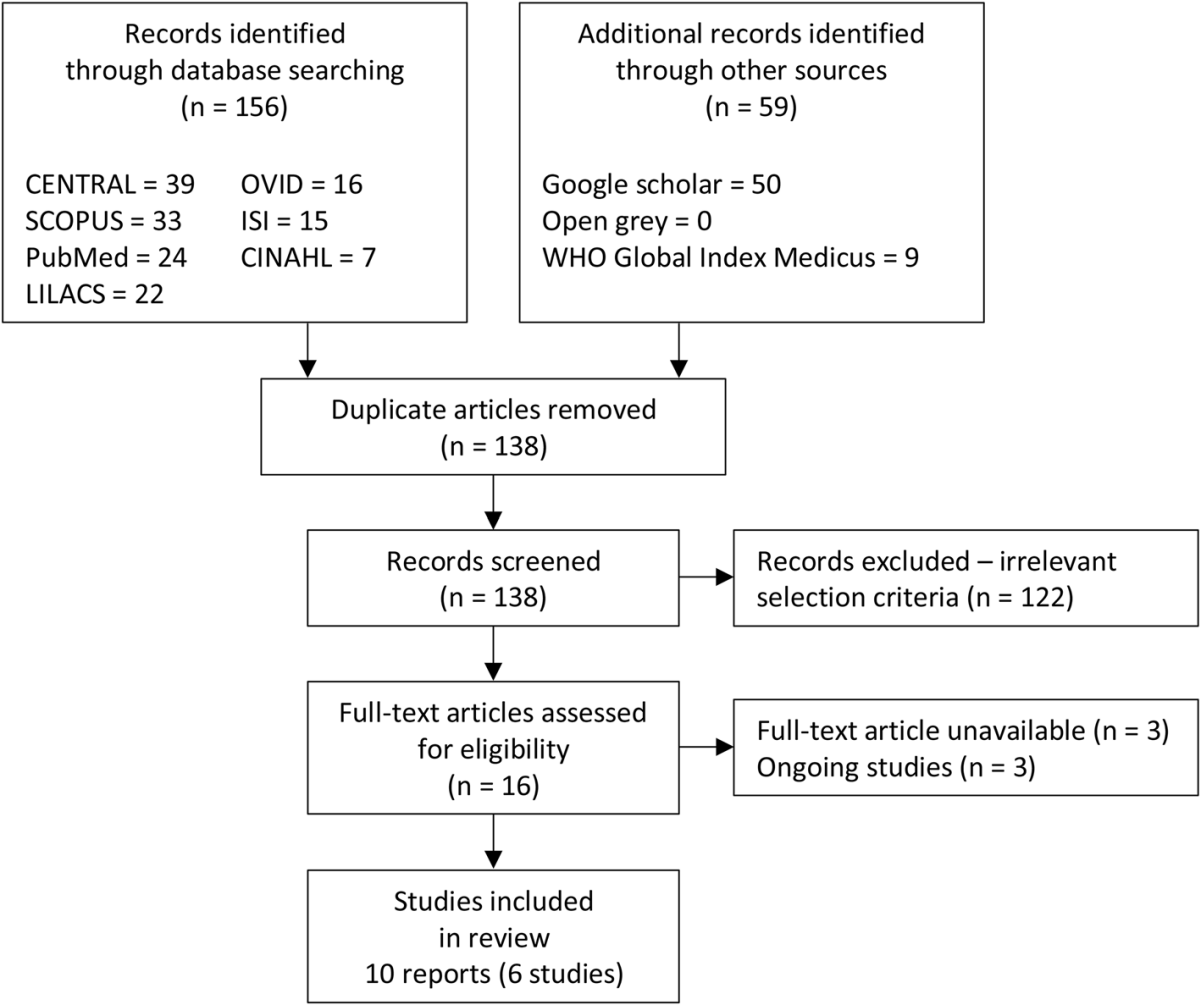
PRISMA diagram.

Figure 1 displays the PRISMA flowchart for study selection. A broad search yielded 156 reports from the electronic database searches. Fifty-nine reports were identified from other sources. After deduplication, 138 reports were screened, and 122 reports that did not meet the inclusion criteria were excluded. After reviewing the full texts of 16 reports that potentially met the review inclusion criteria, six reports were excluded because of unavailable full texts (n = 3) and ongoing studies (n = 3). Ten reports of six studies involving 1707 participants were included in the quantitative synthesis (Supplementary Tables S3–8).

### Characteristics of included studies

Five studies were published in peer-reviewed journals^22–26^. One included study was available only in an academic search engine^27^. Table 1 displays the detailed characteristics of the included studies. Five included studies were undertaken in the United States, whereas the remaining included study was conducted in India. One included study recruited only women who underwent elective CS^24^.

**Table 1:** Characteristics of the included studies.

Author Year (setting)	Intervention	Method of placental delivery	Responsible person for glove changing	Timing of glove change	n	Emergency CS (%)	Ruptured membrane (%)	Routine antibiotic prophylaxis
Atkinson 1996 (USA)	No glove change	Manual	Not applicable	Not applicable	162	80.0	59.0	Received
	No glove change	Spontaneous	Not applicable	Not applicable	164	78.0	59.0	
	Gloves change	Manual	Primary and assistant surgeons	After delivery of the fetus	161	80.0	58.0	
	Gloves change	Spontaneous			156	83.0	52.0	
Cernadas 1998 (USA)	No glove change	Manual	Not applicable	Not applicable	26	46.2	Mixed	Based on physicians and clinical circumstances
	No glove change	Spontaneous	Not applicable	Not applicable	27	44.4		
	Glove change	Manual	Primary surgeons	After delivery of the fetus	27	40.7		
	Glove change	Spontaneous			28	35.7		
Devvoor 2014 (India)	No glove change	Not stated	Not applicable	Not applicable	50	60.0	Mixed	Received
	Glove change	Not stated	Entire surgical team	After delivery of the fetus	50	44.0		
	Glove change	Not stated		After delivery of the placenta	50	68.0		
Scrafford 2018 (USA)	No glove change	Not stated	Not applicable	Not applicable	250	0.0	39.4	Received
	Gloves change	Not stated	Entire surgical team	After delivery of the placenta	236	0.0	42.0	
Turrentine 1996 (USA)	No glove change	Manual	Not applicable	Not applicable	115	49.5	Mixed	Received
	Gloves change	Manual	Not stated	After delivery of the fetus	113	54.9		
Ventolini 2004 (USA)	No glove change	Not stated	Not applicable	Not applicable	46	NR	Intact membrane	Received
	Gloves change	Not stated	Entire surgical team	After delivery of the placenta	46	NR		

N, number of participants; CS, cesarean section; USA, United States; Manual, Manual placental removal method; Spontaneous, Spontaneous placental removal method; NR, not reported.

Of the six included studies, glove change was performed by all surgical team members in three studies^24,26,27^. One included study did not state the person responsible for glove change^25^. In the two remaining included studies, glove change was performed by only the primary surgeon (one study)^23^ or primary and assistant surgeons (one study)^22^.

Glove change was performed after delivery of the fetus and after delivery of the placenta in three and two included studies, respectively^22–26^. In a study by Devvoor et al.^27^, there were three comparison groups: the group without glove change, the group with glove change after delivery of the fetus, and the group with glove change after delivery of the placenta.

The effect of glove change assessed on multiple variables for the same outcome was noted in Devvoor et al.^27^. This study reported three outcomes related to incisional SSI including induration, gaping, and pus. We therefore synthesized incisional SSI using three-level meta-analysis.

Three included studies did not state their applied methods of placental delivery applied^24,26,27^. Prophylactic antibiotics were routinely administered in five included studies^22,24–27^. In one included study, prophylactic antibiotics were selectively given based on the decision of the attending physician^23^ (Table 1).

### Risk of bias in included studies

**Figure 2:**
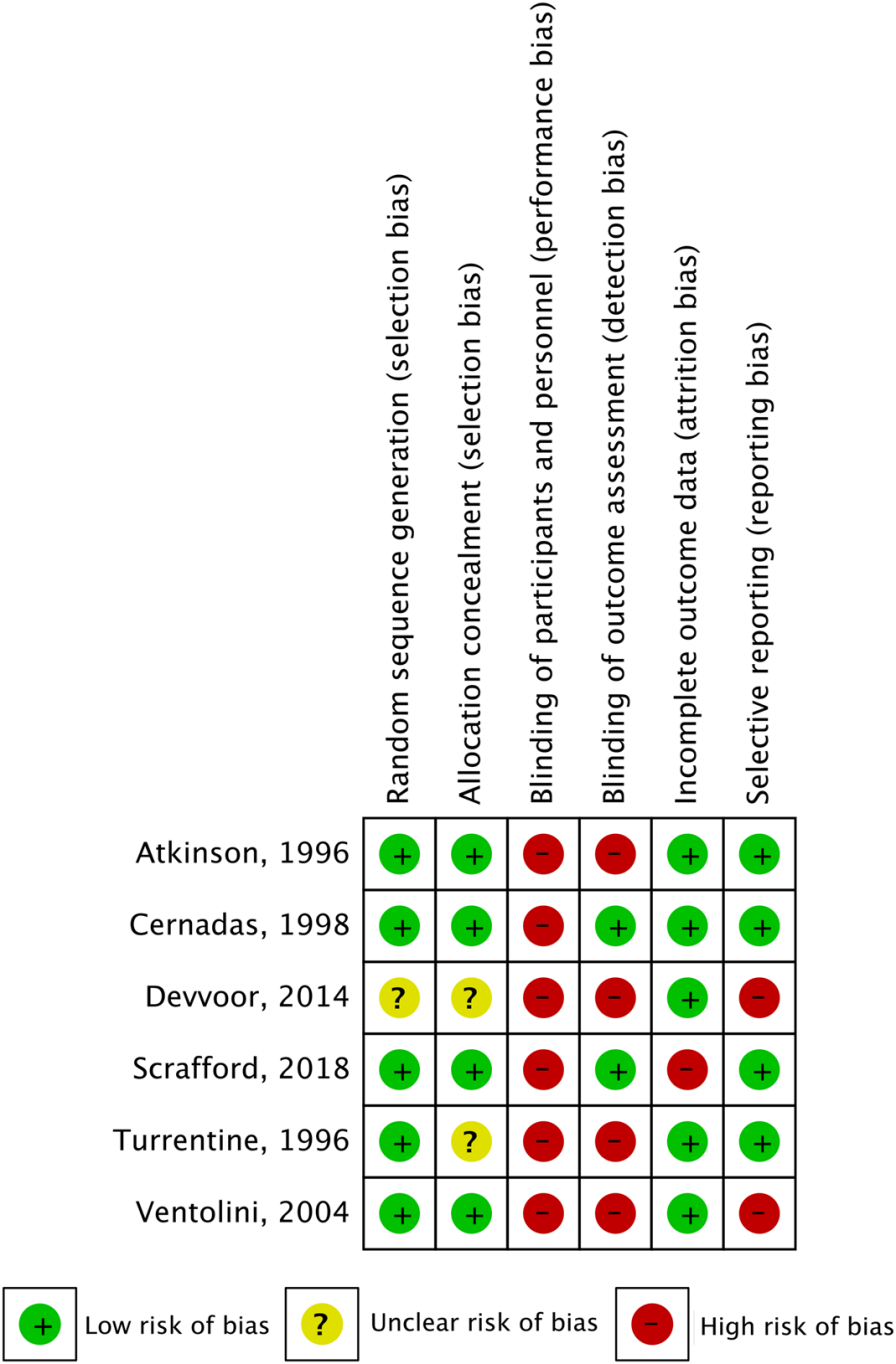
Summary risk of bias of included studies.

Figure 2 shows a summary of the risk of bias for each included study. Two included studies were judged as having an unclear risk of selection bias^25,27^. Because blinding of personnel was technically impossible, all included studies were thus at high risk of performance bias. A high risk of attrition bias and reporting bias was noted in one^24^ and two included studies^26,27^, respectively (Supplementary Table S5).

### Effects of interventions

#### Incidence of endometritis

**Figure 3:**
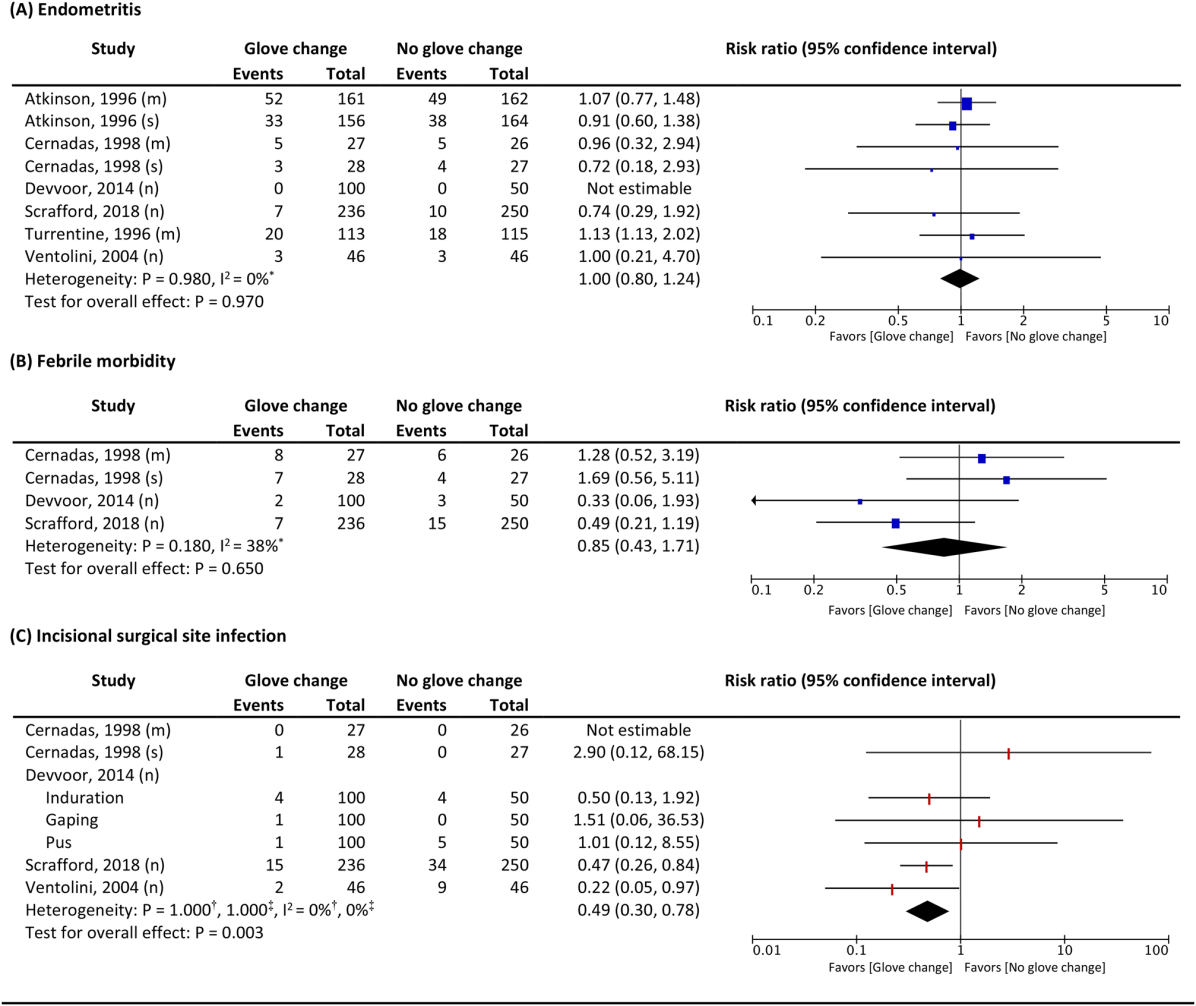
Effects of intervention on (**A**) Endometritis, (**B**) Febrile morbidity, and (**C**) Incisional surgical site infection. (n) = not specified placental delivery method, (m) = manual placental removal, (s) = spontaneous placental removal, ^*^ heterogeneity of two-level meta-analysis explained by I^2^, ^†^heterogeneity of three-level meta-analysis explained by variance within the study ${(\sigma }_{2}^{2})$, ^‡^heterogeneity of three-level meta-analysis explained by variance between the studies $({\sigma }_{3}^{2})$

**Figure 4:**
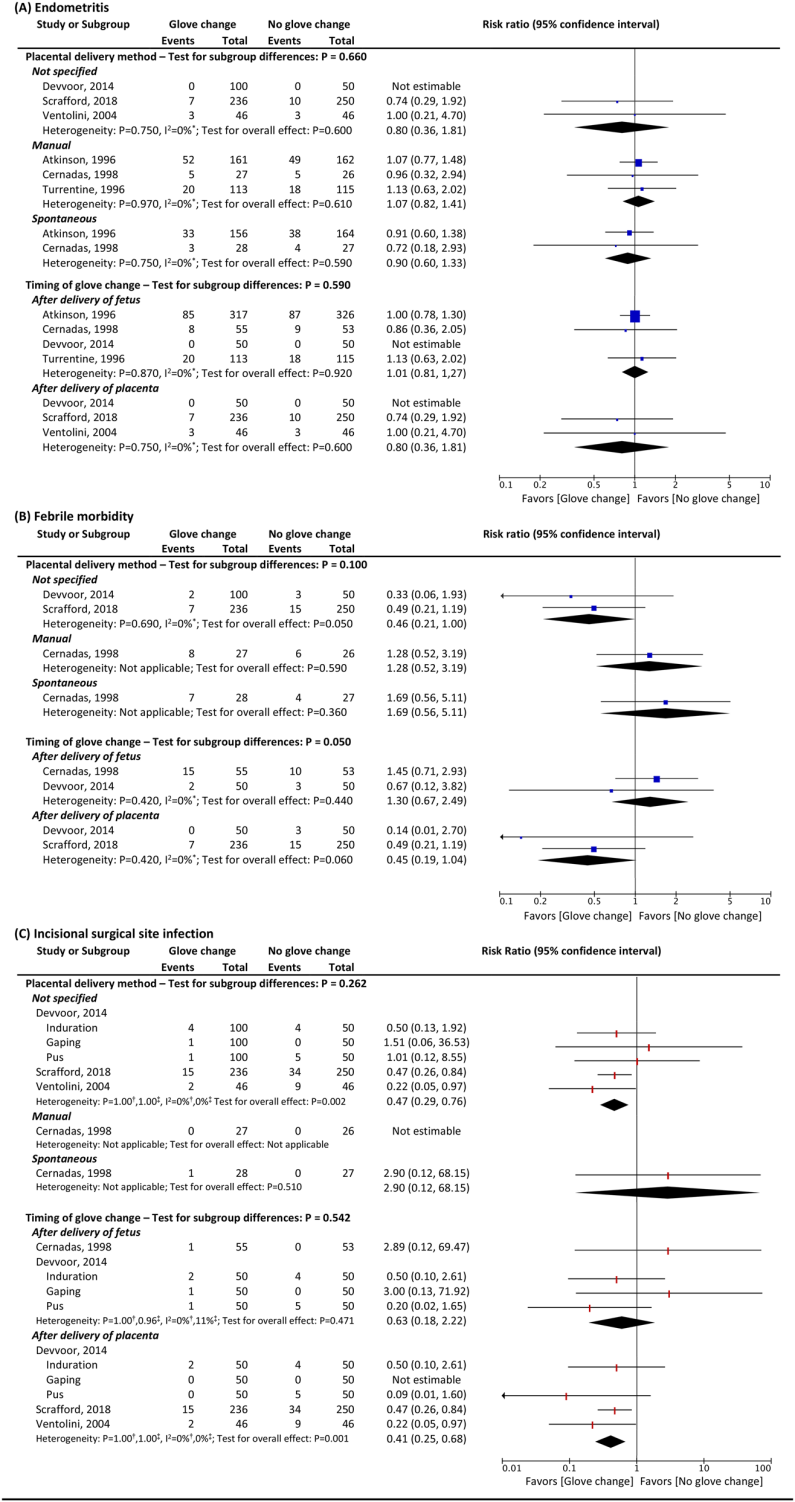
Subgroup analyses of the effects of intervention by placental delivery method and timing of glove change. Manual = manual placental removal, Spontaneous = spontaneous placental delivery, ^*^ heterogeneity of two-level meta-analysis explained by I^2^, ^†^heterogeneity of three-level meta-analysis explained by variance within the study ${(\sigma }_{2}^{2})$, ^‡^heterogeneity of three-level meta-analysis explained by variance between the studies $({\sigma }_{3}^{2})$

Changing gloves during CS did not significantly reduce the risk of endometritis compared to no change in gloves (pooled RR 1.00, 95% CI 0.80, 1.24; six studies; 1707 participants; Fig. 3)^22–27^. Subgroup analyses showed no significant difference in the risk of endometritis between the two comparison groups when stratified by the type of placental delivery method and timing of intraoperative glove change (Fig. 4).

#### Incidence of febrile morbidity

There was no significant difference in the risk of postoperative febrile morbidity between women assigned to the group with intraoperative glove change and those who were assigned to the group without glove change (pooled RR 0.85, 95% CI 0.43, 1.71; three studies; 744 participants; Fig. 3)^23,24,27^. Subgroup analyses indicated no significant benefit of glove change during CS in reducing the risk of postoperative febrile morbidity across the types of placental delivery methods and timing of the glove change (Fig. 4).

#### Incidence of incisional SSI

Overall, intraoperative glove change was associated with a reduction in the risk of incisional SSI (pooled RR 0.49, 95% CI 0.30, 0.78; four studies; 836 participants; Fig. 3)^23,24,26,27^. However, when focused on the included studies that stated their placental delivery methods, the impact of changing gloves on the risk of incisional SSI was inconclusive, as there were only small, included studies with very low event rates contributing to the analysis (Fig. 4). Additional subgroup analysis according to the time of glove change indicated that intraoperative glove change after delivery of the placenta may be preferable to glove change after delivery of the fetus in reducing the risk of incisional SSI (Fig. 4).

### Sensitivity analysis

Sensitivity analyses by excluding studies^25,27^ with a high or unknown risk of selection bias showed no marked difference in terms of the magnitude of associations for all outcomes (Table 2).

**Table 2:** Effects of intervention and sensitivity analysis.

Outcomes	Original analysis			Sensitivity analysis		
	k (n)	Participant	RR (95% CI; p-value)	k (n)	Participant	RR (95% CI; p-value)
**Overall**						
Endometritis	6 (8)	1707	1.00 (0.80, 1.24; p = 0.970)*	4 (6)	1329	0.98 (0.77, 1.23; p = 0.840)*
Febrile morbidity	3 (4)	744	0.85 (0.43, 1.71; p = 0.650)*	2 (3)	594	0.98 (0.46, 2.06; p = 0.950)*
Incisional SSI	4 (7)	836	0.49 (0.30, 0.78; p = 0.003)^†^	3 (4)	686	0.44 (0.23, 0.86; p = 0.020)*
**Subgroup analysis**						
By placental delivery method						
Endometritis						
Not specified	3 (3)	728	0.80 (0.36, 1.81; p = 0.600)*	2 (2)	578	0.80 (0.36, 1.81; p = 0.600)*
Manual	3 (3)	604	1.07 (0.82, 1.41; p = 0.610)*	2 (2)	376	1.06 (0.78, 1.45; p = 0.720)*
Spontaneous	2 (2)	375	0.90 (0.60, 1.33; p = 0.590)*	2 (2)	375	0.90 (0.60, 1.33; p = 0.590)*
Febrile morbidity						
Not specified	2 (2)	636	0.46 (0.21, 1.00; p = 0.050)*	1 (1)	486	0.49 (0.21, 1.19; p = 0.120)
Manual	1 (1)	53	1.28 (0.52, 3.19; p = 0.590)	1 (1)	53	1.28 (0.52, 3.19; p = 0.590)
Spontaneous	1 (1)	55	1.69 (0.56, 5.11; p = 0.360)	1 (1)	55	1.69 (0.56, 5.11; p = 0.360)
Incisional SSI						
Not specified	3 (5)	728	0.47 (0.29, 0.76; p = 0.002)^†^	2 (2)	578	0.42 (0.25, 0.73; p = 0.002)*
Manual	1 (1)	53	Not estimable	1 (1)	53	Not estimable
Spontaneous	1 (1)	55	2.90 (0.12, 68.15; p = 0.510)	1 (1)	55	2.90 (0.12, 68.15; p = 0.510)
By timing of glove change						
Endometritis						
After delivery of fetus	4 (4)	1079	1.01 (0.81, 1.27; p = 0.920)*	2 (2)	751	0.99 (0.78, 1.27; p = 0.950)*
After delivery of placenta	3 (3)	678	0.80 (0.36, 1.81; p = 0.600)*	2 (2)	578	0.80 (0.36, 1.81; p = 0.600)*
Febrile morbidity						
After delivery of fetus	2 (2)	208	1.30 (0.67, 2.49; p = 0.440)*	1 (1)	108	1.45 (0.71, 2.93; p = 0.310)
After delivery of placenta	2 (2)	586	0.45 (0.19, 1.04; p = 0.060)*	1 (1)	486	0.49 (0.21, 1.19; p = 0.120)
Incisional SSI						
After delivery of fetus	2 (4)	208	0.63 (0.18, 2.22; p = 0.471)^†^	1 (1)	108	2.89 (0.12, 69.47; p = 0.510)*
After delivery of placenta	3 (4)	678	0.41 (0.25, 0.68; p = 0.001)^†^	2 (2)	578	0.42 (0.25, 0.73; p = 0.002)*

k, number of studies; n, number of effect sizes; RR, risk ratio; CI, confidence interval; SSI, surgical site infection; Manual, manual placental removal method; Spontaneous, spontaneous placental removal method.

*Two-level meta-analysis; †three-level meta-analysis.

### Quality of evidence

The quality of evidence for endometritis was low due to a lack of information on selection of participants and lack of blinding in the included studies. We graded the quality of evidence for febrile morbidity as very low due to a lack of information on selection of participants and lack of blinding in the included studies and imprecision of estimation. For incisional SSI, we graded the quality of evidence as moderate due to lack of blinding in the included studies.

## Discussion

This study is an assessment of the associations between glove change during CS and postoperative complications, including endometritis, febrile morbidity, and incisional SSI. This systematic review highlights that changing gloves during CS reduced the risk of incisional SSI. Risks of postoperative endometritis and febrile morbidity, however, were not altered by changing gloves. Sensitivity analyses applying the risk of selection bias showed no clinically important changes in the direction of these associations. Interestingly, intraoperative glove change after delivery of the placenta may be preferable to glove change after delivery of the fetus.

Almeida et al.^28^ published their conference proceeding abstract, which was a systematic review investigating the impact of changing gloves during CS on the risks of postoperative complications. The authors concluded that women assigned to undergo changing gloves after delivery of the placenta had a lower incidence of wound infection than those assigned to the control group (RR: 0.34, 95% CI 0.18, 0.65).

Our review, which is based on ten reports of six RCTs involving 1707 unique participants, reaffirmed that changing gloves during CS reduced the risk of incisional SSI with an overall reduction in the rate of 51% (pooled RR 0.49; 95% CI 0.30, 0.78). Our review can provide additional insights into the results of the existing review in that the sensitivity analyses excluding the studies with a high or unclear risk of selection bias underlined the robustness of the results. The characteristics of the included studies, including surgical team members responsible for the glove change, administration of prophylactic antibiotics, method of placental delivery, timing of glove change, reported outcomes, and risk-of-bias assessment, were determined and presented in greater detail by our review. Subgroup analyses that attempted to assess whether methods of placental delivery and timing of the glove change alter the benefit of changing gloves were also performed in our review. To our knowledge, this is the first review to provide the full details of a meta-analysis on this issue. In addition, our review applied a three-level meta-analysis model for the outcomes that involved jointly analyzing several and related variables to minimize an overestimate of the pooled results^15,16^.

Although available evidence notes a reduction in the risk of incisional SSI following changing gloves during CS, the most appropriate timing of the glove change remains inconclusive. A single RCT involving 100 participants that was undertaken to assess the impact of the timing of glove change on postoperative infectious complications noted similar risks of endometritis, postoperative febrile morbidity, and wound complications between a glove change after delivery of the fetus and a glove change after delivery of the placenta^27^. However, a small number of studies with small sample sizes and very low event rates contributed to this finding, thus precluding any meaningful conclusion. It is worth noting that our subgroup analysis according to the timing of the glove change suggested that changing gloves after delivery of the placenta may be preferable to changing gloves after delivery of the fetus (Fig. 4). High-quality RCTs with sufficient sample sizes are required to confirm this interesting finding.

Based on a previous systematic review conducted to provide evidence-based guidance for surgical decisions during CS, recommendations with high-certainty evidence according to the US Preventive Services Task Force included preskin incision prophylactic antibiotics, cephalad-caudad blunt uterine extension, spontaneous placental removal, surgeon preference on uterine exteriorization, single-layer uterine closure when future fertility is undesired, and suture closure of the thick subcutaneous tissue. Glove change during CS, however, was not recommended based on available evidence in this review^29^. In our updated review, changing gloves during CS significantly minimized the risk of incisional SSI with an overall reduction in the incidence of incisional SSI of approximately 50%. The quality of this evidence according to the GRADE approach is moderate. Intraoperative change in gloves, therefore, seems to be a promising intervention to lessen the risk of SSI following CS.

Nevertheless, the limitations of current evidence are worthy of consideration in the interpretation of review findings. First, ample evidence suggests that the methods of placental delivery and infectious complications following CS are related^30–33^. Routine manual removal of the placenta at CS increases postpartum maternal infectious morbidity^30–33^. Information regarding the method of placental delivery, however, was not provided in three of six studies included in this review, which did not enable us to evaluate the impact of the method of placental delivery on the benefits of changing gloves during CS. Second, six records retrieved from the comprehensive search were classified as potentially included studies, but their full texts could not be obtained (three records); additionally, there were ongoing studies awaiting an assessment (three records). Future updated meta-analyses are required when these results are made available. Third, given limited information, evidence regarding whether the type of surgical team member in the operating field who is assigned to change gloves during CS makes a difference in the risk of infectious complications remains unknown. Fourth, subgroup analyses were based on few included studies hence they may have been underpowered to detect differences in the effects of interventions. Finally, the limited number of studies included in this review precluded our ability to assess the potential of small-study effects in this meta-analysis. These limitations should be considered when integrating the policy of glove change during CS into clinical practice.

In conclusion, updated evidence implies that changing gloves during CS can significantly minimize the risk of incisional SSI. Risks of postoperative endometritis and febrile morbidity, however, were not altered by changing gloves. Glove changes after delivery of the placenta may be preferable to glove changes after delivery of the fetus. Any further studies should focus on an assessment of glove change among women undergoing spontaneous placental delivery, which is currently a standard practice^29^.

## Supplementary Information

Supplementary Information.

## Data Availability

The datasets generated and/or analyzed during the current study are not publicly available but could be made available on reasonable request.
