# Author Correction: Changing gloves during cesarean section for prevention of postoperative infections: a systematic review and meta-analysis

**DOI:** 10.1038/s41598-022-26901-9

**Published:** 2023-01-02

**Authors:** Siwanon Rattanakanokchai, Nuntasiri Eamudomkarn, Nampet Jampathong, Bao-Yen Luong-Thanh, Chumnan Kietpeerakool

**Affiliations:** 1 Department of Epidemiology and Biostatistics, Faculty of Public Health, Khon Kaen University, Khon Kaen, 40002 Thailand; 2 Department of Obstetrics and Gynecology, Faculty of Medicine, Khon Kaen University, Khon Kaen, 40002 Thailand; 3 Department of Epidemiology, Biostatistics and Demography, Faculty of Public Health, Hue University of Medicine and Pharmacy, Hue University, 06 Ngo Quyen Street, Hue, 530000 Vietnam

Correction to: *Scientific Reports* https://doi.org/10.1038/s41598-021-84259-w, published online 25 February 2021

The original version of this Article contained an error in the Methods section where the original version of the protocol registered at PROSPERO was not attributed.

“This systematic review was performed and is reported according to the Preferred Reporting Items for Systematic Reviews and Meta-Analyses (PRISMA) statement (Supplementary Table S1)^9^.”

now reads:

“This systematic review was performed and is reported according to the Preferred Reporting Items for Systematic Reviews and Meta-Analyses (PRISMA) statement (Supplementary Table S1)^9^. The protocol for this systematic review was initially designed by the researcher team at Sheffield Teaching Hospitals NHS Trust, UK, and registered at PROSPERO repository on 26 October 2018^10^. The registration page did not include any updates at the time of the onset of our study; we considered the topic important because of the ease and availability of this intervention in clinical setting in LMICs and therefore decided to perform the review using revised meta-analysis methods.”

The protocol is now cited as Reference 10:

10. Almeida, J. R., Narice B, F., Madhuvrata, P. & Farrell, T. Effects of changing gloves during caesarean section: a systematic review and meta-analysis. PROSPERO 2018 CRD42018110529 (Available from: https://www.crd.york.ac.uk/prospero/display_record.php?ID=CRD42018110529)

As a result, the subsequent References have been renumbered.

The original Article has been corrected.

