# Supplementary material for: Changing gloves during cesarean section for prevention of postoperative infections: a systematic review and meta-analysis
Source: Sci Rep. 2021 Feb 25;11:4592. doi: 10.1038/s41598-021-84259-w (PMC7907110; doi:10.1038/s41598-021-84259-w)
Supplement: Supplementary file 1 — Supplementary Information. [file 41598_2021_84259_MOESM1_ESM.pdf]

Supplementary information

Changing gloves during cesarean section for prevention of postoperative infections: a systematic review and meta-analysis

Siwanon Rattanakanokchai[1], Nuntasiri Eamudomkarn[2], Nampet Jampathong[2], Bao-Yen Luong-Thanh[3], Chumnan Kietpeerakool[2,*]

[1] Department of Epidemiology and Biostatistics, Faculty of Public Health, Khon Kaen University, Khon Kaen 40002, Thailand
[2] Department of Obstetrics and Gynecology, Faculty of Medicine, Khon Kaen University, Khon Kaen 40002, Thailand
[3] Department of Epidemiology, Biostatistics and Demography, Faculty of Public Health, Hue University of Medicine and Pharmacy, Hue University,
06 Ngo Quyen Street, Hue, 530000, Vietnam

Supplementary Table S1: PRISMA checklist

			Reported on page#
Title	1	Identify the report as a systematic review, meta-analysis, or both.	1
**ABSTRACT**			
Structured summary	2	Provide a structured summary including, as applicable: background; objectives; data sources; study eligibility criteria, participants, and interventions; study appraisal and synthesis methods; results; limitations; conclusions and implications of key findings; systematic review registration number.	2
**INTRODUCTION**			
Rationale	3	Describe the rationale for the review in the context of what is already known.	3-4
Objectives	4	Provide an explicit statement of questions being addressed with reference to participants, interventions, comparisons, outcomes, and study design (PICOS).	4
**METHODS**			
Protocol and registration	5	Indicate if a review protocol exists, if and where it can be accessed (e.g., Web address), and, if available, provide registration information including registration number.	NA
Eligibility criteria	6	Specify study characteristics (e.g., PICOS, length of follow-up) and report characteristics (e.g., years considered, language, publication status) used as criteria for eligibility, giving rationale.	4-5
Information sources	7	Describe all information sources (e.g., databases with dates of coverage, contact with study authors to identify additional studies) in the search and date last searched.	4-5
Search	8	Present full electronic search strategy for at least one database, including any limits used, such that it could be repeated.	Supplementary information
Study selection	9	State the process for selecting studies (i.e., screening, eligibility, included in systematic review, and, if applicable, included in the meta-analysis).	5
Data collection process	10	Describe method of data extraction from reports (e.g., piloted forms, independently, in duplicate) and any processes for obtaining and confirming data from investigators.	5

Data items	11	List and define all variables for which data were sought (e.g., PICOS, funding sources) and any assumptions and simplifications made.	5-6
Risk of bias in individual studies	12	Describe methods used for assessing risk of bias of individual studies (including specification of whether this was done at the study or outcome level), and how this information is to be used in any data synthesis.	5
Summary measures	13	State the principal summary measures (e.g., risk ratio, difference in means).	6
Synthesis of results	14	Describe the methods of handling data and combining results of studies, if done, including measures of consistency (e.g., I^2) for each meta-analysis.	6

From: Moher D, Liberati A, Tetzlaff J, Altman DG, The PRISMA Group (2009). Preferred Reporting Items for Systematic Reviews and Meta-Analyses: The PRISMA Statement. PLoS Med 6(7): e1000097. doi:10.1371/journal.pmed1000097.

For more information, visit: **www.prisma-statement.org**.

Supplementary Table S2: Description of search strategy of PubMed database

Search	Query
#1	Caesarean section[MH]
#2	(("Caesarean" OR "cesarean" OR "abdominal") AND (section* OR "delivery" OR "deliveries" OR "birth" OR "births")) OR "C-section" OR "C section" OR "C-sections" OR "C sections" OR "CS" OR ("post" AND ("cesarean" OR "caesarean"))
#3	#1 OR #2
#4	surgical gloves[MH]
#5	protective gloves[MH]
#6	"glove" OR "gloves"
#7	#4 OR #5 OR #6
#8	"change" OR "changes" OR "changing" OR "renewal" OR "renewed" OR "over"
#9	#3 AND #7 AND #8

Supplementary Table S3: Reports that cannot obtain full-text and ongoing studies

Reports that cannot obtain full text
1. Keating, A., Lanbers, D., Khoury, J. & Siddiqi, T. Reduction of post-cesarean infectious morbidity by changes of gloves prior to operative closure. in American journal of obstetrics and gynecology vol. 176 S58 (1997).
2. Keating, A., Lanbers, D., Khoury, J. & Siddiqi, T. Reduction of post-cesarean infectious morbidity by change of gloves prior to operative closure. in Acta Diabetologica Latina vol. 176 (1997).
3. Szatmary, P. F. et al. Intraoperative glove change and assisted spontaneous placental delivery as methods to decrease the risk of post-caesarean endometritis. Magy. Noorv. Lapja 67, 225–232 (2004).
**Ongoing studies**
1. CTRI/2019/01/017110. Evaluation of surgical site infection bundle to decrease infectious in women undergoing caesarean section. http//www.who.int/trialsearch/Trial2.aspx?TrialID=CTRI/2019/01/017110 (first received. 16 January 2019) (2019).
2. NCT03500055. Abdominal Closure Bundle to Reduce Surgical Site Infection in Patients Undergoing C-sections. Cesarean Section Lead to Reduction in Surgical Site Infection? - A Randomized Controlled Trial. https//clinicaltrials.gov/show/NCT03500055 (first received: 17 April 2018) (2018).
3. NCT04006067. The Impact of Changing Gloves During Cesarean Section on Post-operative Wound Complication. *https://clinicaltrials.gov/show/NCT04006067 (first received: 2 July 2019)*.

Supplementary Table S4: Reports of the included studies

**Atkinson 1996**
1. Atkinson, M., Wren, A., Owen, J. & Hauth, J. Intraoperative glove change and spontaneous placental delivery reduces post-cesarean endometritis. *Am. J. Obstet. Gynecol.* **172**, 300 (1995).
2. * Atkinson, M., Owen, J., Wren, A. & Hauth, J. The Effect of Manual Removal of the Placenta on Post-Cesarean Endometritis. *Obstet. Gynecol.* **87**, 99–102 (1996).
**Cernadas 1998**
3. Cernadas, M., Smulian, J. C., Giannina, C. & Ananth, C. V. The effects of method of placental delivery and intraoperative glove changing on post-cesarean febrile morbidity. *Acta Diabetol. Lat.* **176**, (1997).
4. * Cernadas, M., Smulian, J. C., Giannina, G. & Ananth, C. V. Effects of placental delivery method and intraoperative glove changing on postcesarean febrile morbidity. *J. Matern. Fetal. Med.* **7**, 100–104 (1998).
**Devoor 2014**
5. Devoor, A. K., Roopadevi & Manjunath, G. H. Effects of Intraoperative 'Changing Glove Technique' on Post Cesarean Infectious Morbidity. *Sch. J. Appl. Med. Sci.* **2**, 3118–3122 (2014).
**Scrafford 2018**
6. NCT02907892. Effect of Intra-operative Glove-changing During Cesarean Section on Post-op Complications: A Randomized Controlled Trial. *https//clinicaltrials.gov/ct2/show/NCT02907892 (first received: 16 Sept. 2016)*.
7. Reddy, B. & Scrafford, J. Effect of Intra-Operative Glove-Changing During Cesarean on Post-Op Complications. *Obstet. Gynecol.* **129**, 4S-5S (2017).
8. * Scrafford, J. D., Reddy, B., Rivard, C. & Vogel, R. I. Effect of intra-operative glove changing during cesarean section on post-operative complications: a randomized controlled trial. *Arch. Gynecol. Obstet.* **297**, 1449-1454 (2018).
**Turrentine 1996**
9. Turrentine, M. A. & Banks, T. A. Effect of Changing Gloves Before Placental Extraction on Incidence of Postcesarean Endometritis. *Infect. Dis. Obstet. Gynecol.* **4**, 16–19 (1996).
**Ventolini 2014**
10. Ventolini, G., Neiger, R. & McKenna, D. Decreasing Infectious Morbidity in Cesarean Delivery by Changing Gloves. *J. Reprod. Med.* **49**, 13–16 (2004).

* The primary reference for the study

Supplementary Table S5: Judgement for risk of bias assessment using the Cochrane Risk of Bias Tool for Randomized Controlled Trials*

**Bias**	**Authors' judgement**	**Support for judgement**
**Atkinson, 1996**		
Random sequence generation (selection bias)	Low risk	Using computer-generated number
Allocation concealment (selection bias)	Low risk	Using sealed, opaque envelop
Blinding of participants and personnel (performance bias)	High risk	Blinding of personnel was technical impossible and this may affect the outcome.
Blinding of outcome assessment (detection bias)	High risk	Not mentioned. Detection bias is a very important issue in this type of study and thus may influence an assessment of outcome.
Incomplete outcome data (attrition bias)	Low risk	Outcome completely assessed
Selective reporting (reporting bias)	Low risk	Outcome completely reported as per the objective
**Cernadas, 1998**		
Random sequence generation (selection bias)	Low risk	Using computer-generated random group assignments
Allocation concealment (selection bias)	Low risk	Using sealed envelope containing computer-generated random numbers
Blinding of participants and personnel (performance bias)	High risk	Blinding of personnel was technical impossible and this may affect the outcome.
Blinding of outcome assessment (detection bias)	Low risk	Outcome assessor was unaware about the allocation
Incomplete outcome data (attrition bias)	Low risk	Outcome completely assessed
Selective reporting (reporting bias)	Low risk	Outcome completely reported as per the objective
**Devvoor, 2014**		
Random sequence generation (selection bias)	Unclear risk	No statement
Allocation concealment (selection bias)	Unclear risk	No statement
Blinding of participants and personnel (performance bias)	High risk	Blinding of personnel was technical impossible and this may affect the outcome.
Blinding of outcome assessment (detection bias)	High risk	Not mentioned. Detection bias is a very important issue in this type of study and thus may influence an assessment of outcome.
Incomplete outcome data (attrition bias)	Low risk	Outcome completely assessed
Selective reporting (reporting bias)	High risk	Data regarding detailed characteristics of wound infection was separately reported
**Scrafford, 2018**		
Random sequence generation (selection bias)	Low risk	A computer-generated allocation card from a stack placed within an envelope
Allocation concealment (selection bias)	Low risk	A computer-generated allocation card from a stack placed within an envelope
Blinding of participants and personnel (performance bias)	High risk	Blinding of personnel was technical impossible and this may affect the outcome.
Blinding of outcome assessment (detection bias)	Low risk	Outcome assessor was unaware about the allocation
Incomplete outcome data (attrition bias)	High risk	High default rates across the comparison groups
Selective reporting (reporting bias)	Low risk	Outcome completely reported as per the objective

**Bias**	**Authors' judgement**	**Support for judgement**
**Turrentine, 1996**		
Random sequence generation (selection bias)	Low risk	Using computer-generated number
Allocation concealment (selection bias)	Unclear risk	No statement
Blinding of participants and personnel (performance bias)	High risk	Blinding of personnel was technical impossible and this may affect the outcome.
Blinding of outcome assessment (detection bias)	High risk	Not mentioned. Detection bias is a very important issue in this type of study and thus may influence an assessment of outcome.
Incomplete outcome data (attrition bias)	Low risk	Outcome completely assessed
Selective reporting (reporting bias)	Low risk	Outcome completely reported as per the objective
**Ventolini, 2004**		
Random sequence generation (selection bias)	Low risk	Using computer-generated random group assignments
Allocation concealment (selection bias)	Low risk	Using sealed envelope containing computer-generated random numbers
Blinding of participants and personnel (performance bias)	High risk	Blinding of personnel was technical impossible and this may affect the outcome.
Blinding of outcome assessment (detection bias)	High risk	Not mentioned. Detection bias is a very important issue in this type of study and thus may influence outcome assessment
Incomplete outcome data (attrition bias)	Low risk	Outcome completely assessed
Selective reporting (reporting bias)	High risk	No data regarding febrile morbidity which was stated as one of the outcomes of interest

***** *Cochrane Handbook for Systematic Reviews of Interventions version 6.0 (updated July 2019)*. *Cochrane Collaboration* (2019)

Supplementary Table S6: Summary of reported outcomes of the included studies

**Author, year**	**Outcomes**			
	Endometritis	Febrile morbidity	Incisional surgical site infection	Total
Atkinson, 1996	✓			1
Cernadas, 1998	✓	✓	✓	3
Devvoor, 2014	✓	✓	✓	3
Scrafford, 2018	✓	✓	✓	3
Turrentine, 1996	✓			1
Ventolini, 2004	✓		✓	3
Total	6	3	4	

Supplementary Table S7: Details of reported outcomes

Author, year (method of placental delivery)	Glove change		No glove change		Risk ratio	(95% CI)
	Events	Total	Events	Total		
**Overall**						
**Endometritis**						
Atkinson, 1996 (manual)	52	161	49	162	1.07	(0.77, 1.48)
Atkinson, 1996 (spontaneous)	33	156	38	164	0.91	(0.60, 1.38)
Cernadas, 1998 (manual)	5	27	5	26	0.96	(0.32, 2.94)
Cernadas, 1998 (spontaneous)	3	28	4	27	0.72	(0.18, 2.93)
Devvoor, 2014 (not specified)	0	100	0	50	Not estimable	
Scrafford, 2018 (not specified)	7	236	10	250	0.74	(0.29, 1.92)
Turrentine, 1996 (manual)	20	113	18	115	1.13	(1.13, 2.02)
Ventolini, 2004 (not specified)	3	46	3	46	1.00	(0.21, 4.70)
**Febrile morbidity**						
Cernadas, 1998 (manual)	8	27	6	26	1.28	(0.52, 3.19)
Cernadas, 1998 (spontaneous)	7	28	4	27	1.69	(0.56, 5.11)
Devvoor, 2014 (not specified)	2	100	3	50	0.33	(0.06, 1.93)
Scrafford, 2018 (not specified)	7	236	15	250	0.49	(0.21, 1.19)
**Incision surgical site infection**						
Cernadas, 1998 (manual)	0	27	0	26	Not estimable	
Cernadas, 1998 (spontaneous)	1	28	0	27	2.90	(0.12, 68.15)
Devvoor, 2014 (not specified)						
Induration	4	100	4	50	0.50	(0.13, 1.92)
Gaping	1	100	0	50	1.51	(0.06, 36.53)
Pus	1	100	5	50	1.01	(0.12, 8.55)
Scrafford, 2018 (not specified)	15	236	34	250	0.47	(0.26, 0.84)
Ventolini, 2004 (not specified)	2	46	9	46	0.22	(0.05, 0.97)
**Author, year**	**Glove change**		**No glove change**		**Risk ratio**	**(95% CI)**
	**Events**	**Total**	**Events**	**Total**		
**Subgroup analysis by placental delivery method**						
**Endometritis**						
**Not specified method of placental delivery**						
Devvoor, 2014	0	100	0	50	Not estimable	
Scrafford, 2018	7	236	10	250	0.74	(0.29, 1.92)
Ventolini, 2004	3	46	3	46	1.00	(0.21, 4.70)
**Manual placental delivery**						
Atkinson, 1996	52	161	49	162	1.07	(0.77, 1.48)
Cernadas, 1998	5	27	5	26	0.96	(0.32, 2.94)
Turrentine, 1996	20	113	18	115	1.13	(1.13, 2.02)
**Spontaneous placental delivery**						
Atkinson, 1996	33	156	38	164	0.91	(0.60, 1.38)
Cernadas, 1998	3	28	4	27	0.72	(0.18, 2.93)
**Febrile morbidity**						
**Not specified method of placental delivery**						
Devvoor, 2014	2	100	3	50	0.33	(0.06, 1.93)
Scrafford, 2018	7	236	15	250	0.49	(0.21, 1.19)
**Manual placental delivery**						
Cernadas, 1998	8	27	6	26	1.28	(0.52, 3.19)
**Spontaneous placental delivery**						
Cernadas, 1998	7	28	4	27	1.69	(0.56, 5.11)

Author, year	Glove change		No glove change		Risk ratio	(95% CI)
	Events	Total	Events	Total		
**Incision surgical site infection**						
**Not specified method of placental delivery**						
Devvoor, 2014						
Induration	4	100	4	50	0.50	(0.13, 1.92)
Gaping	1	100	0	50	1.51	(0.06, 36.53)
Pus	1	100	5	50	1.01	(0.12, 8.55)
Scrafford, 2018	15	236	34	250	0.47	(0.26, 0.84)
Ventolini, 2004	2	46	9	46	0.22	(0.05, 0.97)
**Manual placental delivery**						
Cernadas, 1998	0	27	0	26	Not estimable	
**Spontaneous placental delivery**						
Cernadas, 1998	1	28	0	27	2.90	(0.12, 68.15)
**Subgroup analysis by timing of glove change**						
**Endometritis**						
**After delivery of fetus**						
Atkinson, 1996	85	317	87	326	1.00	(0.78, 1.30)
Cernadas, 1998	8	55	9	53	0.86	(0.36, 2.05)
Devvoor, 2014	0	50	0	50	Not estimable	
Turrentine, 1996	20	113	18	115	1.13	(0.63, 2.02)
**After delivery of placenta**						
Devvoor, 2014	0	50	0	50	Not estimable	
Scrafford, 2018	7	236	10	250	0.74	(0.29, 1.92)
Ventolini, 2004	3	46	3	46	1.00	(0.21, 4.70)
**Febrile morbidity**						
**After delivery of fetus**						
Cernadas, 1998	15	55	10	53	1.45	(0.71, 2.93)
Devvoor, 2014	2	50	3	50	0.67	(0.12, 3.82)
**After delivery of placenta**						
Devvoor, 2014	0	50	3	50	0.14	(0.01, 2.70)
Scrafford, 2018	7	236	15	250	0.49	(0.21, 1.19)
**Incision surgical site infection**						
**After delivery of fetus**						
Cernadas, 1998	1	55	0	53	2.89	(0.12, 69.47)
Devvoor, 2014						
Induration	2	50	4	50	0.50	(0.10, 2.61)
Gaping	1	50	0	50	3.00	(0.13, 71.92)
Pus	1	50	5	50	0.20	(0.02, 1.65)
**After delivery of placenta**						
Devvoor, 2014						
Induration	2	50	4	50	0.50	(0.10, 2.61)
Gaping	0	50	0	50	Not estimable	
Pus	0	50	5	50	0.09	(0.01, 1.60)
Scrafford, 2018	15	236	34	250	0.47	(0.26, 0.84)
Ventolini, 2004	2	46	9	46	0.22	(0.05, 0.97)

Abbreviation: Manual: Manual placental removal method, Spontaneous: Spontaneous placental removal method.

Supplementary Table S8: Details of reported outcomes stratified by delivery methods and timing of glove change

Author, year	Intervention	Method/Timing	Outcome	Event	Total number
**Placental delivery method**					
**Atkinson, 1996**	No glove change	Manual	Endometritis	49	162
	No glove change	Spontaneous	Endometritis	38	164
	Gloves change	Manual	Endometritis	52	161
	Gloves change	Spontaneous	Endometritis	33	156
**Cernadas, 1998**	No glove change	Manual	Endometritis	5	26
			Febrile morbidity	6	26
			Incisional SSI	0	26
	No glove change	Spontaneous	Endometritis	4	27
			Febrile morbidity	4	27
			Incisional SSI	0	27
	Glove change	Manual	Endometritis	5	27
			Febrile morbidity	8	27
			Incisional SSI	0	27
	Glove change	Spontaneous	Endometritis	3	28
			Febrile morbidity	7	28
			Incisional SSI	1	28
**Devvoor, 2014**	No glove change	Not stated	Endometritis	0	50
			Febrile morbidity	3	50
			Incisional SSI		
			Induration	4	50
			Gaping	0	50
			Pus	5	50
	Glove change	Not stated	Endometritis	0	50
			Febrile morbidity	2	50
			Incisional SSI		
			Induration	4	100
			Gaping	1	100
			Pus	1	100
**Scrafford, 2018**	No glove change	Not stated	Endometritis	10	250
			Febrile morbidity	15	250
			Incisional SSI	34	250
	Gloves change	Not stated	Endometritis	7	236
			Febrile morbidity	7	236
			Incisional SSI	15	236
**Turrentine, 1996**	No glove change	Manual	Endometritis	18	115
	Gloves change	Manual	Endometritis	20	113
**Ventolini, 2004**	No glove change	Not stated	Endometritis	3	46
			Febrile morbidity	Not reported	
			Incisional SSI	9	46
	Gloves change	Not stated	Endometritis	3	46
			Febrile morbidity	Not reported	
			Incisional SSI	2	46
**Timing of glove change**					
**Atkinson, 1996**	No glove change	Not applicable	Endometritis	87	326
	Gloves change	After delivery of the fetus	Endometritis	85	317
**Cernadas, 1998**	No glove change	Not applicable	Endometritis	9	53
			Febrile morbidity	10	53
			Incisional SSI	0	53
	Glove change	After delivery of the fetus	Endometritis	8	55
			Febrile morbidity	15	55
			Incisional SSI	1	55
**Devvoor, 2014**	No glove change	Not applicable	Endometritis	0	50
			Febrile morbidity	3	50
			Incisional SSI		
			Induration	4	50
			Gaping	0	50
			Pus	5	50

Author, year	Intervention	Method/Timing	Outcome	Event	Total number
**Devvoor, 2014** (cont.)	Glove change	After delivery of the fetus	Endometritis	0	50
			Febrile morbidity	2	50
			Incisional SSI		
			Induration	2	50
			Gaping	1	50
			Pus	1	50
	Glove change	After delivery of the placenta	Endometritis	0	50
			Febrile morbidity	0	50
			Incisional SSI		
			Induration	2	50
			Gaping	0	50
			Pus	0	50
**Scrafford, 2018**	No glove change	Not applicable	Endometritis	10	250
			Febrile morbidity	15	250
			Incisional SSI	34	250
	Gloves change	After delivery of the placenta	Endometritis	7	236
			Febrile morbidity	7	236
			Incisional SSI	15	236
**Turrentine, 1996**	No glove change	Not applicable	Endometritis	18	115
	Gloves change	After delivery of the fetus	Endometritis	20	113
**Ventolini, 2004**	No glove change	Not applicable	Endometritis	3	46
			Febrile morbidity	Not reported	
			Incisional SSI	9	46
	Gloves change	After delivery of the placenta	Endometritis	3	46
			Febrile morbidity	Not reported	
			Incisional SSI	2	46

Abbreviation: Manual: Manual placental removal method, Spontaneous: Spontaneous placental removal method, SSI: surgical site infection.
